# Supplementary material for: Dual Inhibition of EGFR and IGF-1R Signaling Leads to Enhanced Antitumor Efficacy against Esophageal Squamous Cancer
Source: Int J Mol Sci. 2022 Sep 8;23(18):10382. doi: 10.3390/ijms231810382 (PMC9499412; doi:10.3390/ijms231810382)
Supplement: Supplementary file 1 [file ijms-23-10382-s001.zip › ijms-1887793-supplementary.pdf]

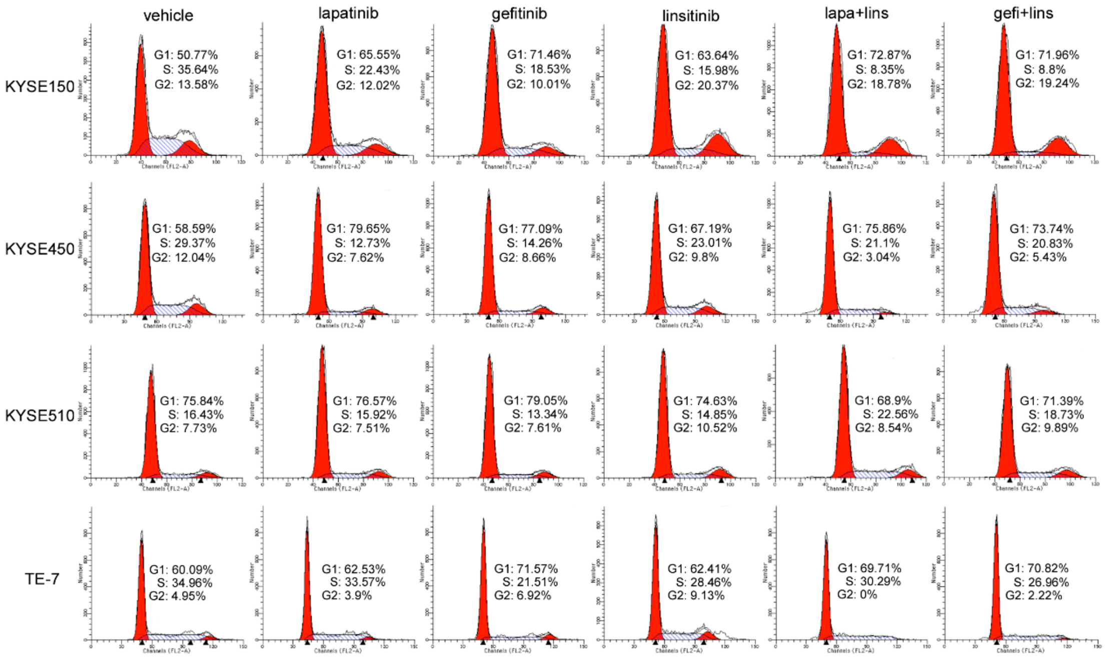

Supplementary Figure S1. The flow cytometry data of the effects of lapatinib, gefitinib, linsitinib alone or in combination on the cell cycle distribution. Four ESCC cells were stained with propidium iodide (PI) after treatment by lapatinib, gefitinib, linsitinib, lapatinib+linsitinib and gefitinib+linsitinib for 48 h. The fluorescence intensity was measured by flow cytometer and cells distributed in G1, S and G2/M phase were analyzed by ModiFit software.

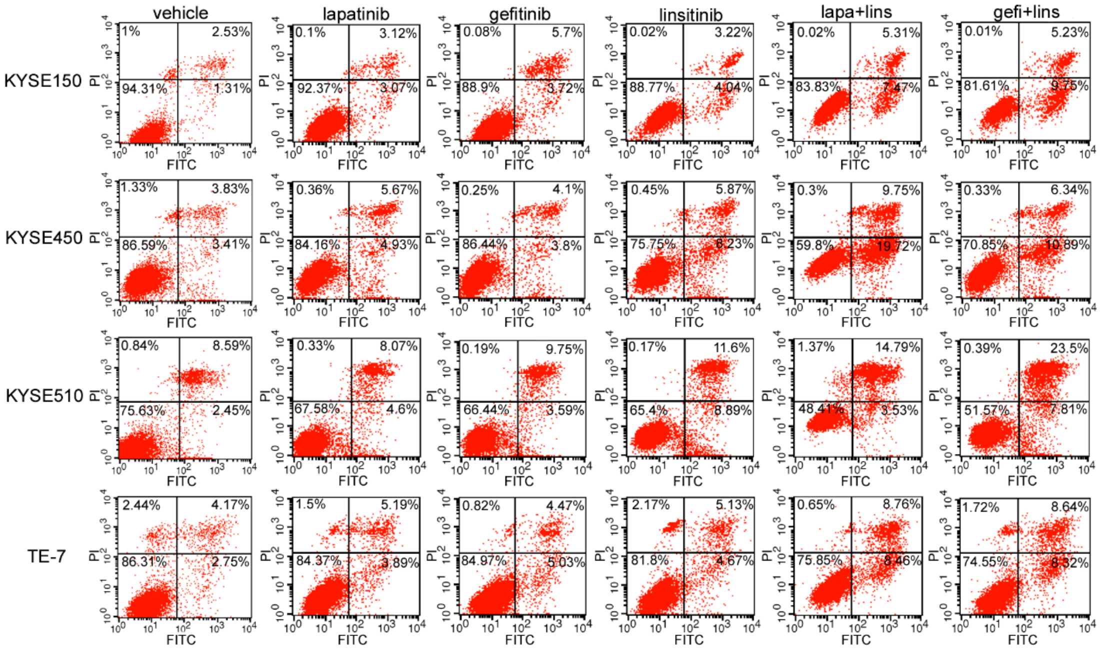

Supplementary Figure S2. The flow cytometry data of the effects of lapatinib, gefitinib, linsitinib alone or in combination on cell apoptosis. ESCC cells were exposed to single drugs or in combinations as indicated, and then the apoptotic cells were stained with Annexin V-FITC and PI. The fluorescence intensity was measured by flow cytometer. The lower left quadrant (FITC^{-} PI^{-}) indicated the live cells, the lower right quadrant (FITC^{+} PI^{-}) indicated the early apoptosis cells, the upper right quadrant (FITC^{+} PI^{+}) indicated the late apoptosis cells, and the upper left quadrant (FITC^{-} PI^{+}) indicated the dead cells. The ratios of cell apoptosis were calculated by the sum of early apoptosis cells and the late apoptosis cells.

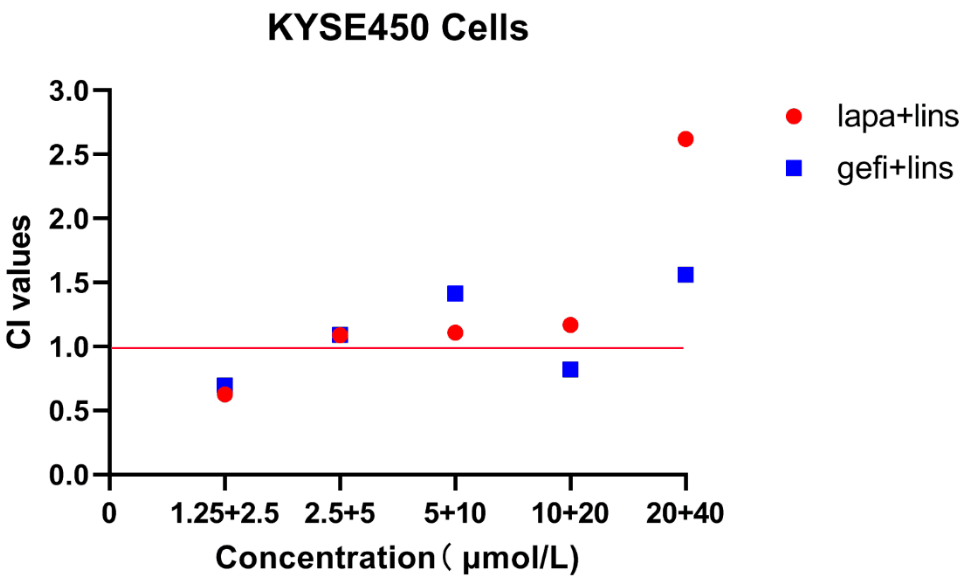

Supplementary Figure S3. The CI values of lapatinib or gefitinib in combination with linsitinib at ratio of 1:2 against KYSE450 cells, calculated by using the Chou–Talalay method.

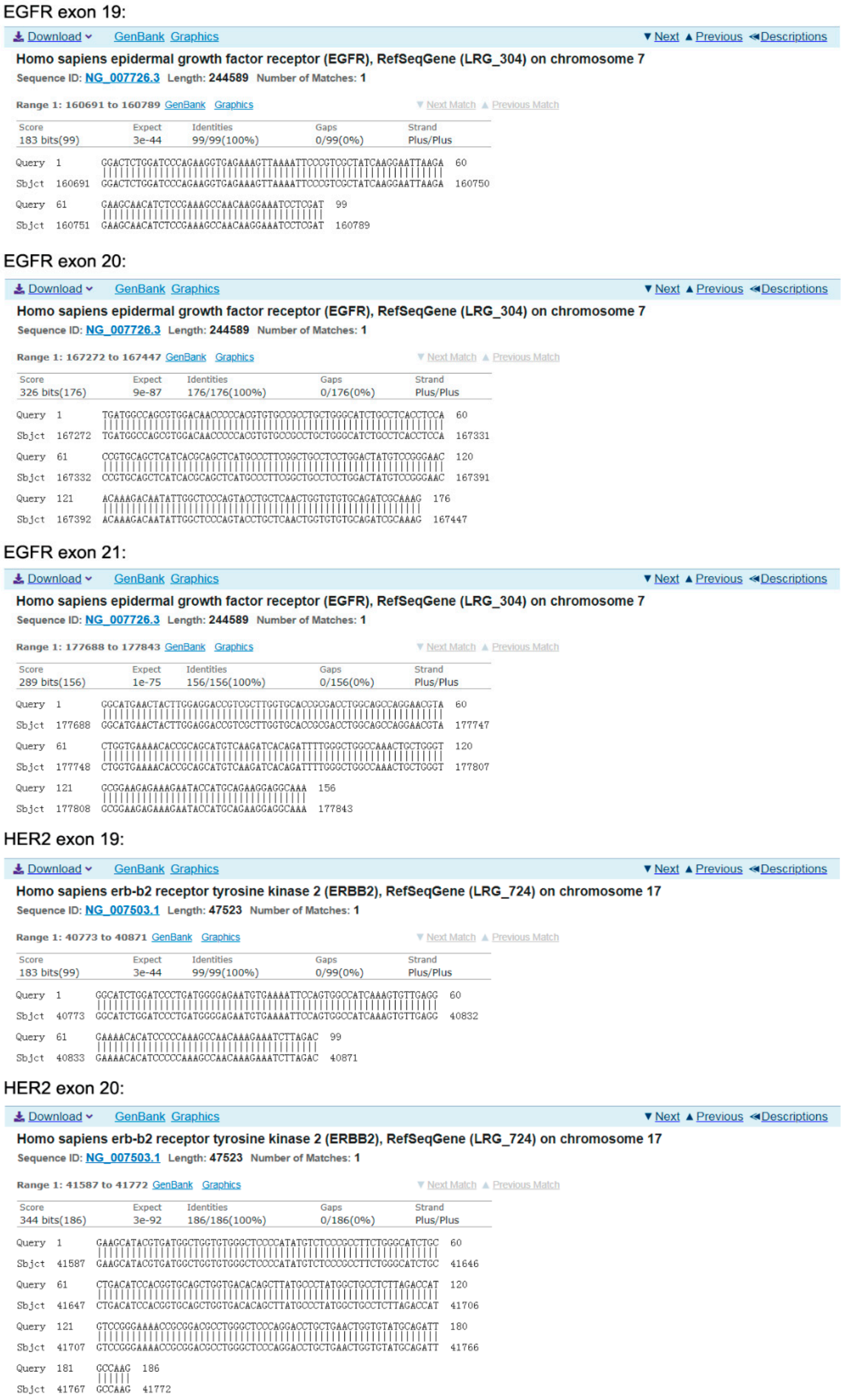

Supplementary Figure S4. Sequence alignment of EGFR exon 19, 20, 21 and HER2 exon 19, 20 from KYSE450 cells to the *Homo sapiens* epidermal growth factor receptor (EGFR) gene and *Homo sapiens* erb-b2 receptor tyrosine kinase 2 (ERBB2) gene, respectively, by using BLAST.
